# Medium-term and long-term renal function changes with direct oral anticoagulants in elderly patients with atrial fibrillation

**DOI:** 10.3389/fphar.2023.1210560

**Published:** 2023-07-04

**Authors:** Giuseppe Armentaro, Graziella D’Arrigo, Mario Bo, Velia Cassano, Sofia Miceli, Annalisa Pitino, Giovanni Tripepi, Santina Maria Grazia Romeo, Giorgio Sesti, Gregory Y. H. Lip, Daniele Pastori, Mercedes Gori, Angela Sciacqua

**Affiliations:** ^1^ Department of Medical and Surgical Sciences, University Magna Græcia of Catanzaro, Catanzaro, Italy; ^2^ Institute of Clinical Physiology (IFC-CNR), Section of Reggio Calabria, Reggio Calabria, Italy; ^3^ Section of Geriatrics, Department of Medical Sciences, University of Turin, Molinette Hospital, Turin, Italy; ^4^ Institute of Clinical Physiology (IFC-CNR), Section of Rome, Rome, Italy; ^5^ Department of Advanced Biomedical Sciences, University “Federico II”, Naples, Italy; ^6^ Department of Clinical and Molecular Medicine, University Rome-Sapienza, Rome, Italy; ^7^ Department of Clinical MedicineAalborg University, Liverpool Centre for Cardiovascular Science, University of Liverpool, Liverpool John Moores University and Liverpool Heart and Chest Hospital, Liverpool, Denmark; ^8^ Department of Clinical Internal, Anesthesiological and Cardiovascular Sciences, Sapienza University of Rome, Rome, Italy

**Keywords:** atrial fibrillation, chronic kidney disease, elderly, DOACs, warfarin-like drugs, EGFR decline

## Abstract

**Objective:** Atrial Fibrillation (AF) and chronic kidney disease frequently coexist in the elderly. Warfarin-like drugs (WLDs) may be associated with a relatively greater decrease of estimated glomerular filtration rate (eGFR) as compared to direct oral anticoagulants (DOACs), but there is no evidence on the medium- and long-term changes. To further elucidate this issue in elderly patients with AF, we investigated the renal function deterioration in the two groups of the study (DOACs or WLDs).

**Patients and Methods:** A total of 420 AF patients were enrolled (mean age: 77.0 ± 6.0 years; 136 on WLDs and 284 on DOACs). These patients underwent three eGFR measurements during the follow-up period. The between-arms difference of eGFR decline over time was investigated by Linear Mixed Models and group-based trajectory model analyses.

**Results:** In the whole study cohort, after a median follow-up of 4.9 years (interquartile range: 2.7–7.0 years), eGFR decreased from 67.4 ± 18.2 to 47.1 ± 14.3 mL/min/1.73 m^2^ (*p* < 0.001). Remarkably, patients on DOACs experienced a significantly smaller eGFR decline than WLDs patients (−21.3% vs. −45.1%, *p* < 0.001) and this was true both in the medium-term (−6.6 vs. −19.9 mL/min/1.73 m^2^) and in the long-term (−13.5 *versus* −34.2 mL/min/1.73 m^2^) period. After stratification into five subgroups according to trajectories of renal function decline over time, logistic regression showed that DOACs patients had from 3.03 to 4.24-fold greater likelihood to belong to the trajectory with less marked eGFR decline over time than WLDs patients.

**Conclusion:** Elderly patients with AF on treatment with DOACs had a relatively smaller decline of eGFR over time compared to those on treatment with WLDs. This is consistent with what was partly reported in the literature.

## 1 Introduction

Atrial fibrillation (AF) affects approximately 5% of the over-65 population (Miyasaka Y., et al., 2006; Hindricks et al., 2021). Chronic kidney disease (CKD) is a frequent complication in the elderly that shares several risk factors with AF and also increases the risk of *de novo* AF (Watanabe H., et al., 2009; Bansal N., et al., 2013). CKD *per se* increases the risk of thromboembolism, bleeding, and mortality in AF patients (Potpara T.S., et al., 2020), whereas the new onset of AF is associated with an increased risk (+80%) of renal function decline (Ha J.T., et al., 2019). Oral anticoagulant therapy (OAT), particularly direct oral anticoagulants (DOACs) which are preferred to Warfarin-like drugs (WLDs), is the cornerstone for the primary and secondary prevention of stroke and systemic embolism in patients with AF, including those with mild to moderate CKD. DOACs consist of Dabigatran which is a direct thrombin inhibitor and factor X inhibitors: Apixaban, Rivaroxaban, and Edoxaban. However, WLDs inhibit the production of biologically active vitamin K-dependent clotting factors via the inhibition of vitamin K epoxide reductase; this enzyme converts vitamin K epoxide to vitamin K hydroquinone, the latter being required for the gamma-carboxylation and activation of several clotting factors (Menichelli D., et al., 2021; Roberti R., et al., 2021; Steffel J., et al., 2021). In the general population, the physiological reduction of estimated glomerular filtration rate (eGFR) is expected to be −0.55 and −0.33 mL/min/1.73 m^2^/year in men and women, respectively (Halbesma N., et al., 2006). Greater declines in eGFR were reported in elderly patients with AF (Chen T.H., et al., 2022) and this reduction seemed to be influenced by the use of different OATs. Several studies suggest a more favorable effect of DOACs compared with WLDs with regard to renal function decline and adverse outcomes in patients with AF in the short term (Yao X., et al., 2017; Pastori D., et al., 2020; Lee W.C., et al., 2021; Sitticharoenchai P., et al., 2021; Batra G., et al., 2022). However, the ROCKET-AF and ARISTOTLE trials, and several real-world studies, show no difference between the two OATs on renal function decline (Bohm M., et al., 2015; Fordyce C.B., et al., 2016; Hijazi Z., et al., 2016; Fauchier L., et al., 2018; Lee W.C., et al., 2021; Pérez A.G., et al., 2022; Trevisan M., et al., 2022). To comprehensively investigate this topic, in a single-center prospective cohort study on elderly patients with AF, we aimed to: 1) assess the effect of DOACs vs. WLDs on absolute and relative renal function decline over time; 2) test whether the use of DOACs was associated with slower (i.e., less steep) trajectories of renal function decline over time as compared to those of WLDs-treated patients.

## 2 Methods

### 2.1 Study setting, design, and sample

Between January 2008 and October 2019, all consecutive outpatients with AF referred to the Geriatrics Department, “Magna Graecia” University of Catanzaro, Italy, were enrolled. Inclusion criteria were non-valvular AF, age ≥65 years, availability of at least 1 year of follow-up, and informed consent to take part in the study. Exclusion criteria were valvular diseases (mechanical prosthetic heart valves or moderate-severe mitral stenosis), previous anticoagulant therapy (thus prevalent-users were excluded), chronic infectious diseases (i.e., human immunodeficiency virus infection, hepatitis C virus, and hepatitis B virus), or autoimmune systemic diseases, active cancer, and liver failure (e.g., cirrhosis). The local Ethical Committee (Comitato Etico Regione Calabria “Area Centro”) approved the protocol (protocol number 2012.63). The procedures used in this study adhere to the tenets of the Declaration of Helsinki. Informed written consent was obtained from all participants.

### 2.2 Clinical variables at baseline and follow up

At enrolment and during the two follow-up visits, all patients underwent a comprehensive medical history, physical examination, and routine electrocardiography as well as took measurements of anthropometric and hemodynamic variables such as weight, height, body mass index, systolic and diastolic blood pressure, and heart rate. Relevant comorbidities and the number and type of drug therapies were also recorded. The risk of ischemic stroke and bleeding was assessed using validated tools for AF patients (Hindricks et al., 2021).

The following indicators of renal function were collected during visits: creatinine levels (Jaffè method, Peake and Whitnig. 2006) and glomerular filtration rate (eGFR) as estimated by CKD-EPI (Chronic Kidney Disease Epidemiology Collaboration) equation (Levey A.S., et al., 2009). All patients had three measurements of eGFR: one at baseline and two during the follow-up period. The first-time window lasted 3.2 ± 2.0 years (medium period) and the second time lasted 6.7 ± 2.3 years (long period).

### 2.3 Sample size calculation

The main objective of the study was to investigate the effect of DOACs vs. WLDs on renal function deterioration over time. Two regression models were applied: 1) the linear mixed model and 2) the multinomial logistic regression model. There were 74 repeated observations for each covariate in the multiple linear mixed models and seven observations for each covariate in the multiple logistic regression model thus guaranteeing an adequate balance between covariates and observations (Peduzzi P., et al., 1996).

### 2.4 Statistical analysis

Normally distributed data were summarized as mean and standard deviation and non-normally distributed data were summarized as median and interquartile range (IQR). Categorical variables were summarised as percent frequencies (%). Between-group comparisons (i.e., WLDs and DOACs treated patients) were carried out using Student’s t-test (for normally distributed data), Mann-Whitney *U*-test (for non-normally distributed data), or χ^2^ test (for binary and categorical data), as appropriate (Altman D.G., 1991).

#### 2.4.1 Linear mixed model and group-based trajectory model analyses

The between-group (WLDs and DOACs) differences of eGFR decline over time between WLDs and DOACs-treated patients were investigated using two statistical techniques: 1) the Linear Mixed Models (LMM) and 2) the Group-Based Trajectory Model (GBTM) analyses.

By LMMs analysis, we investigated the effect of WLDs *versus* DOACs on continuous values of eGFR measurements over time. In this analysis, repeated measurements of eGFR were considered dependent variables, and treatment (WLDs vs. DOACs), time, and treatment-by-time interaction were considered key independent variables (Verbeke G. and Molenberghs G., 2000).

By GBTM analysis (Nagin D.S., 1999), patients were grouped according to the trajectories of their eGFR values over time. The trajectory analysis identified groups with similar eGFR trajectories based on maximum likelihood estimates. Each patient was assigned to the trajectory on the basis of the higher posterior probability. The results of the trajectory were represented using the trajectory plot (including the 95% CIs as well as the percentage of patients in each group). Trajectory groups were ranked according to the eGFR loss over time (from the lowest to the highest). Once trajectories were identified, multinomial crude and adjusted logistic regression models were fitted to assess the relationship between the treatment (WLDs vs. DOACs) and trajectory groups. In this analysis, the reference group was that with the greatest loss of eGFR over time. In these models, data were expressed as probability ratio, 95% Cis, and *p*-value (further details about GBTM analysis are given in the Appendix). In multiple models, we adjusted for all variables that significantly differed between the two treatment groups with the exception of age, hypertension, and previous ACS (because these variables were already included in the score most commonly utilized to predict thromboembolic risk in atrial fibrillation, comprehensive of “congestive heart failure, hypertension, age ≥75 (doubled), diabetes, stroke (doubled), vascular disease, age of 65–74, and sex category (female)” (CHA2DS2VASc) score, i.e., a covariate into the same models). A *p*-value ≤ 0.05 was considered statistically significant. All analyses were performed in the STATA package (16.1 for Windows, TX United States).

## 3 Results

Among 761 patients admitted during the study period, 122 were excluded because they were <65 years old, 97 were on anticoagulant therapy before enrolment, 65 had a mechanical prosthetic valve, 32 had moderate-severe mitral stenosis, 10 had cancer, 8 had liver cirrhosis, and 7 had chronic inflammatory diseases. Therefore, 420 elderly patients were enrolled (see Table 1). Their mean age was 77 ± 6 years, 55% were men, and 9% were habitual smokers. The median CHA_2_DS_2_VASc score was four (IQR: 3–5) and the “Hypertension, Abnormal Renal/Liver Function, Stroke, Bleeding History or Predisposition, Labile INR, Elderly, and Drugs/Alcohol Concomitantly” (HAS-BLED) score was three (IQR: 2–3). In the study sample, 284 patients (67.6%) were treated with DOACs (144 were taking rivaroxaban, 55 dabigatran, 50 apixaban, and 35 edoxaban) and the remaining 136 (32.4%) were on WLDs (110 were taking warfarin and 26 were taking acenocoumarol). Compared with WLDs-treated patients, those receiving DOACs were older, had higher CHA_2_DS_2_VASc scores, and displayed a higher prevalence of several comorbidities and lower renal function at baseline. Moreover, patients on DOACs were also more frequently treated with digoxin, angiotensin receptor neprilysin inhibitor (ARNI), insulin, sodium-glucose cotransporter 2 inhibitor (SGLT2i), and had lower hemoglobin compared to those on WLDs (Table 1).

**TABLE 1:** Baseline characteristics of patients that completed the study among the treatment groups.

	Whole population (N = 420)	WLDs (N = 136)	DOACs (N = 284)	*p*
Demographic and clinical parameters				
Age, *years*	77 ± 6	73 ± 6	78 ± 5	<0.001
Gender (males), *%*	55%	60%	53%	0.22
BMI, *Kg/m* ^*2*^	29 ± 4	30 ± 3	29 ± 4	0.36
Waist, *cm*	109 ± 10	109 ± 8	109 ± 11	0.85
Smokers, *%*	9%	10%	8%	0.54
Systolic BP, *mmHg*	133 ± 12	133 ± 10	132 ± 12	0.63
Diastolic BP, *mmHg*	77 ± 10	77 ± 9	77 ± 10	0.88
Pulse Pressure, *mmHg*	56 ± 12	56 ± 11	56 ± 12	0.73
Atrial Fibrillation, type, *%*				0.66
Paroxysmal	17%	19%	17%	
Persistent	17%	15%	18%	
Permanent	66%	66%	66%	
PM or ICD, *%*	9%	10%	9%	0.80
CHA_2_DS_2_VASc, *pt*	4 (3–5)	4 (3–5)	4 (4–6)	0.001
HAS-BLED, *pt*	3 (2–3)	3 (2–3)	3 (2–3)	0.107
Comorbidities				
Hypertension, *%*	90%	82%	94%	<0.001
Diabetes, *%*	40%	40%	41%	0.82
Dyslipidemia, *%*	44%	37%	47%	0.04
Respiratory insufficiency/COPD, *%*	40%	29%	45%	0.002
Heart failure, *%*	34%	23%	39%	0.002
SAS, *%*	28%	24%	30%	0.15
Type of SAS, *% (n = 118)*				0.61
OSA	40%	47%	37%	
CSA	52%	47%	53%	
MSA	8%	6%	9%	
Severity of SAS, *% (n = 118)*				0.81
Mild	97%	97%	98%	
Moderate	3%	3%	2%	
Severe	0%	0%	0%	
Liver disease, *%*	21%	21%	21%	0.90
Cardiovascular background comorbidities				
Previous Stroke or TIA, *%*	11%	7%	12%	0.12
Previous ACS, *%*	24%	17%	28%	0.02
Vasculopaties, *%*	61%	60%	60%	0.90
Drugs				
PPIs, *%*	91%	90%	91%	0.80
Nitrates, *%*	7%	5%	8%	0.23
ACEi/ARBs, *%*	81%	85%	80%	0.22
β-blockers, *%*	71%	66%	74%	0.12
Digoxin, *%*	21%	12%	26%	0.002
Calcium-blockers, *%*	15%	16%	15%	0.71
MRAs, *%*	25%	21%	27%	0.23
ARNI, *%*	33%	23%	38%	0.002
Anti-Arrhythmics Drugs, *%*	17%	21%	14%	0.08
Metformina/OADs, *%*	23%	23%	23%	0.92
Insulin, *%*	12%	7%	15%	0.03
SGLT2i, *%*	31%	23%	35%	0.015
Incretins, *%*	40%	40%	40%	0.95
Anti-platelet drugs, %	8%	9%	4%	0.08
Lipid-lowering drugs, *%*	49%	51%	49%	0.68
Biochemical parameters				
Albumin, *g/dl*	3.90 ± 0.35	3.89 ± 0.36	3.91 ± 0.35	0.48
Total cholesterol, *mg/dl*	168 ± 44	169 ± 42	168 ± 45	0.83
LDL cholesterol, *mg/dl*	101 ± 35	101 ± 35	101 ± 35	0.99
HDL cholesterol, *mg/dl*	52 ± 18	53 ± 16	52 ± 19	0.71
Triglycerides, *mg/dl*	98 (72–137)	94 (70–133)	100 (72–140)	0.40
Na, *mmol/l*	140.64 ± 2.31	140.88 ± 2.19	140.53 ± 2.36	0.14
Haemoglobin, *g/dl*	13.31 ± 1.53	13.68 ± 0.88	13.13 ± 1.74	<0.001
K, *mmol/l*	4.41 ± 0.37	4.43 ± 0.38	4.40 ± 0.37	0.44
Creatinine, *mg/dl*	1.00 ± 0.28	0.91 ± 0.23	1.05 ± 0.29	<0.001
e-GFR, *ml/min/1.73m2*	67.36 ± 18.16	75.81 ± 17.22	63.32 ± 17.20	<0.001
NT- proBNP, *pg/ml*	562 (512–1,460)	598 (523–1804)	562 (489–1,432)	0.30
Fasting glucose, *mg/dl*	103 (96–116)	106 (99–112)	102 (93–126)	0.54
Fasting Insulin, *µU/ml*	16.36 ± 6.53	15.69 ± 5.89	16.69 ± 6.81	0.14
HOMA,	4.25 (2.96–5.72)	3.88 (2.83–5.25)	4.34 (3.10–6.11)	0.04
Uric acid, *mg/dl*	5.61 ± 0.96	5.58 ± 0.95	5.62 ± 0.96	0.65
AST, *IU/L*	21.17 ± 7.72	19.64 ± 5.43	21.90 ± 8.51	0.005
ALT, *IU/L*	20.65 ± 10.69	19.57 ± 10.05	21.18 ± 10.96	0.15
Alkaline phosphatase, *IU/L*	78 (66–110)	75 (66–99)	78 (66–111)	0.38
GGT, *IU/L*	33 (21–44)	33 (19–44)	32 (21–44)	0.63

Abbreviation: WLDs, Warfarin-like drugs; DOACs, direct oral anticoagulants; BMI, body mass index; BP, blood pressure; PM, pacemaker; ICD, implantable cardioverter defibrillator; COPD, chronic obstructive pulmonary disease; SAS, sleep apnea syndrome; OSA, obstructive sleep apnea; CSA, central sleep apnea; MSA, mixed sleep apnea; TIA, transient ischemic attack; ACS, acute coronary syndrome; PPIs, Proton pump inhibitors; ACEi, angiotensin-converting enzyme inhibitor; ARBs, angiotensin receptor blockers; MRAs, mineralocorticoid receptor antagonists; ARNI, angiotensin receptor neprilysin inhibitor; OADs, oral antidiabetic drugs; SGLT2i, sodium-glucose cotransporter 2 inhibitors; LDL, low-density lipoprotein; HDL, high-density lipoprotein; Na, Sodium; K, potassium; e-GFR, estimated glomerular filtration rate; NT-proBNP, N-terminal pro-brain natriuretic peptide; HOMA, homeostatic model assessment; AST, aspartate transaminase; ALT, alanine aminotransferase; GGT, gamma glutamyltranspeptidase.

### 3.1 Changes of eGFR over time and trajectory analysis

During the follow-up period (median: 4.9 years, IQR: 2.7–7.0 years), all patients underwent three longitudinal measurements of eGFR (see Methods), resulting in a total of 1,260 repeated assessments of eGFR for the data analysis. The individual changes in eGFR in the population and the treatment groups are shown graphically in Supplementary Figure S1. Overall, in the whole study group, eGFR decreased from 67.4 ± 18.2 mL/min/1.73 m^2^ to 47.1 ± 14.3 mL/min/1.73 m^2^ (percentage decrease: 30%, *p* < 0.001) and either absolute or relative declines were significantly smaller in patients on DOACs (from: 63.3 ± 17.2 mL/min/1.73 m^2^ to 49.8 ± 14.6 mL/min/1.73 m^2^; percentage decrease: 21.3%, *p* < 0.001) than in those on WLDs (from: 75.8 ± 17.2 mL/min/1.73 m^2^ to 41.6 ± 11.7 mL/min/1.73 m^2^, percentage decrease: 45.1%, *p* < 0.001).

In the LMM analysis, the effect of DOACs vs. WLDs on renal function decline was significantly heterogeneous (*p* < 0.001) according to time. Overall, the mean eGFR decreased during follow-up (baseline: 67 ± 18 mL/min/1.73 m^2^, follow-up visit 1: 56 ± 15 mL/min/1.73 m^2^, and follow-up visit 2: 47 ± 14 mL/min/1.73 m^2^).

### 3.2 eGFR deterioration for DOACs *versus* WLDs

eGFR decline markedly differed between the two groups (WLDs baseline: 75 ± 17 mL/min/1.73 m^2^, follow-up visit 1: 56 ± 15 mL/min/1.73 m^2^, and follow-up visit 2: 42 ± 12 mL/min/1.73 m^2^; DOACs: baseline: 63 ± 17 mL/min/1.73 m^2^, follow-up visit 1: 57 ± 15 mL/min/1.73 m^2^, and follow-up visit 2: 50 ± 15 mL/min/1.73 m^2^). The between-drugs differences of eGFR deterioration were relatively larger during the long-term period (6.7 ± 2.3 years) [DOACs vs. WLDs: 13.5 (95% CI: 16.1 to −11.0) vs. −34.2 (95% CI: 37.9 to −30.5) between-drugs difference: 20.7 mL/min/1.73 m^2^], compared to that during the medium-term period (3.2 ± 2.0 years) [DOACs vs. WLDs: 6.6 (95% CI: 9.1 to −4.0) vs. −19.9 (95% CI: 23.6 to −16.2), between-drugs difference: 13.3 mL/min/1.73 m^2^]. These results did not materially change after data adjustment for potential confounders, including baseline eGFR (data not shown). No effect of modification by sex was found.

### 3.3 Trajectories analysis of eGFR decline

**FIGURE 1:**
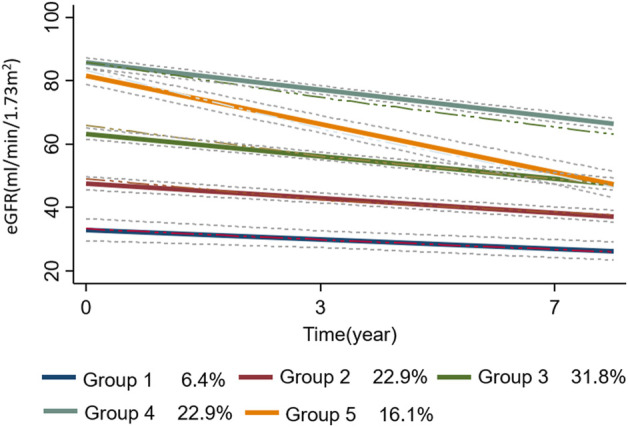
the trajectories (n = 5) of eGFR evolution over time are plotted as a function of time. The dotted lines represent the 95% confidence intervals of each trajectory. In the bottom panel of the figure, the percentage of patients in each group-based trajectory is also reported. Abbreviation: eGFR, estimated glomerular filtration rate.

To identify the best fitting trajectories, a BIC value for each model (Table 2) was considered, starting with a 2-group quadratic order (2 2) for the trajectory shapes. Even though the five trajectories model with all quadratic order (2 2 2 2 2) had a slightly higher BIC (−4922.9) than the linear one (1 1 1 1 1) (−4930.19), we used the last one as a compromise between statistics and clinical judgment (Nagin D.S. 2005). Thus, a five-trajectory model for the eGFR evolution over time was adopted (Figure 1). Six percent of patients were in the first trajectory (lowest decline, −0.99 ± 0.28 mL/min/1.73 m^2^/over 3.3 years), 23% in the second (−1.54 ± 0.18 mL/min/1.73 m^2^/over 3.3 years), 32% in the third (−2.36 ± 0.21 mL/min/1.73m^2^/over 3.3 years), 23% in the fourth (−2.85 ± 0.15 mL/min/1.73m^2^/over 3.3 years), and the remaining 16% in the fifth (greatest decline, −5.06 ± 0.29 mL/min/1.73 m^2^/over 3.3 years) (see Figure 1).

**TABLE 2:** BIC for eGFR according to the number of groups and trajectory shapes and entropy.

Number of groups	Trajectory shapes	BIC^a^ (N = 1,260)	BIC^b^ (N = 420)	Entropy
2	2 2	−5073.82	−5069.43	0.79
2	1 1	−5072.43	−5069.13	0.80
3	2 2 2	−4974.15	−4967.56	0.84
3	1 2 1	−4967.7	−4962.21	0.84
3	1 1 1	−4974.4	−4969.45	0.84
4	2 2 2 2	−4963.95	−4955.16	0.82
4	1 2 2 1	−4956.85	−4949.16	0.82
4	1 1 2 1	−4955.26	−4948.12	0.82
4	1 1 1 1	−4962.82	−4956.23	0.82
5	2 2 2 2 2	−4933.89	−4922.9	0.83
5	1 1 1 1 1	−4938.42	−4930.19	0.81

In Trajectory shapes: 1 = linear; 2 = quadratic.

^a^ BIC, Bayesian information criterion (for the total number of observations).

^b^ BIC, Bayesian information criterion (for the total number of participants).

**FIGURE 2:**
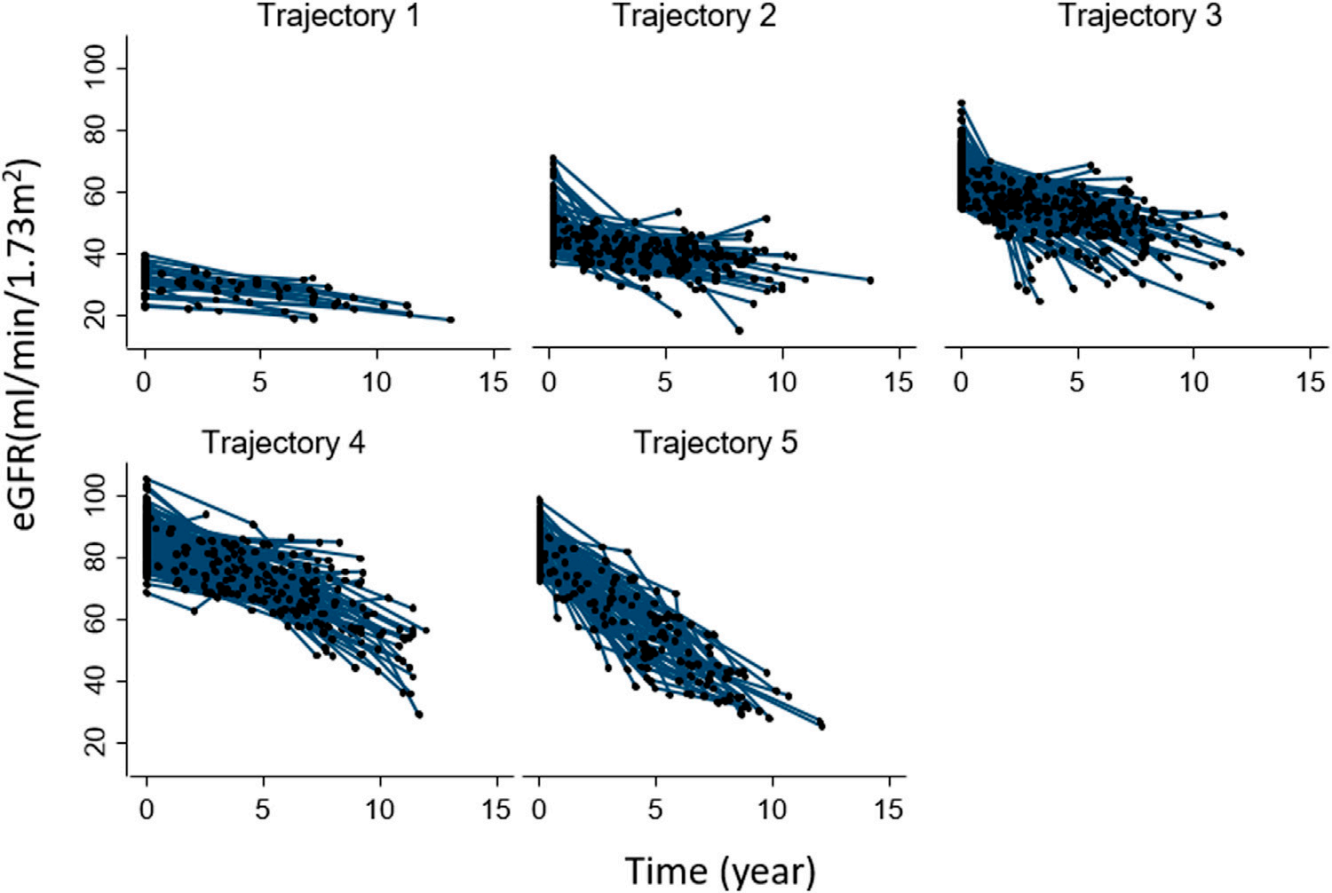
Individual evolution of eGFR over time in each group-based trajectory. Abbreviation: eGFR, estimated glomerular filtration rate.

Patients’ characteristics at baseline according to trajectories are reported in Supplementary Table S3. Patients in the lowest trajectory were older, with a higher proportion being women. Of note, the proportion of patients on DOACs was highest among those in the first trajectory (80%) (i.e., in those having the smallest eGFR decline over time) and decreased in close parallelism with the magnitude of renal function deterioration as assessed by the trajectory analysis (2nd trajectory: 77%, 3rd:74%, 4th: 59%, and 5th: 49%). This result indicates that the larger the proportion of DOACs-treated patients, the smaller the magnitude of eGFR deterioration over time. The individual evolutions over time of eGFR according to each trajectory are shown in Figure 2.

The mean posterior individual probability ranged from 0.79 to 0.82, suggesting overall good discrimination over the follow-up period (>0.70). The odds of correct classification (OCC) was higher than five, indicating that all the trajectory satisfied the optimal model fit criteria (Supplementary Tables S1,2). In Table 2, the values of entropy are given.

### 3.4 Multinomial logistic analysis

The multinomial logistic analysis, comparing the trajectories with the one having the largest decline of eGFR (5th, reference category), indicated that patients on DOACs had a probability to belong to a trajectory associated with a lower renal function deterioration, which was from 3.03 to 4.24 times higher than those on treatment with WLDs (*p* < 0.001) (Table 3). In this model, the fourth and fifth trajectories did not differ between them (*p* = 0.170) (Table 3).

**TABLE 3:** Crude and adjusted multinomial logistic regression analyses

	Crude Analysis	Adjusted analysis*
	*Probability ratio, (95% CI, P) to belong to 1* ^*st*^ *rather than to the 5* ^*th*^ *trajectory*	*Probability ratio (95% CI) to belong to 1st rather than to the 5* ^*th*^ *trajectory*
DOACs versus WLDs	4.24 (1.43-12.61), p=0.009	4.96 (1.47-16.74), p=0.01
	*Probability ratio (95% CI, P) to belong to 2* ^*nd*^ *rather than to the 5* ^*th*^ *trajectory*	P*robability ratio (95% CI) to belong to 2* ^*nd*^ *rather than to the 5th trajectory*
DOACs versus WLDs	3.57 (1.82-6.99), p<0.001	3.83 (1.82-8.07), p<0.001
	*Probability ratio (95% CI, P) to belong to 3rd rather than to the 5* ^*th*^ *trajectory*	Probability ratio (95% CI) to belong to 3^rd^ rather than to the 5^th^ trajectory
DOACs versus WLDs	3.03 (1.64-5.59), p<0.001	3.40 (1.72-6.71), p<0.001
	*Probability ratio (95% CI) to belong to 4th rather than to the 5* ^*th*^ *trajectory*	Probability ratio (95% CI) to belong to 4^th^ rather than to the 5^th^ trajectory
DOACs versus WLDs	1.55 (0.83-2.90), p=0.170	1.94 (0.96-3.91), p=0.065

* Adjusted for baseline characteristics: CHA2DS2VASc score, dyslipidemia, respiratory insufficiency, digoxin, insulin, ARNI, SGLT2i, hemoglobin, HOMA, AST.

The multiple logistic regression model, adjusted for variables that significantly differed between the two treatments, provided similar results (Table 3). In particular, the adjusted probability of patients treated with DOACs belonging to groups with more favorable outcomes (i.e., with a relatively smaller eGFR decline) was 3.40–4.96 times higher than those treated with WLDs (*p* ranging from <0.001 to 0.01, Table 3). In this adjusted model, the comparison between the fourth and fifth trajectories was not significant (*p* = 0.065) (Table 3). Overall, eGFR decreased during follow-up (baseline: 67 ± 18 mL/min/1.73 m^2^, follow-up 1: 56 ± 15 mL/min/1.73 m^2^, and follow-up 2: 47 ± 14 mL/min/1.73 m^2^). Of note, eGFR decline differed between the two treatment groups (WLDs baseline: 75 ± 17 mL/min/1.73 m^2^, follow-up 1: 56 ± 15 mL/min/1.73 m^2^, and follow-up 2: 42 ± 12 mL/min/1.73 m^2^; DOACs: baseline: 63 ± 17 mL/min/1.73 m^2^, follow-up 1: 57 ± 15 mL/min/1.73 m^2^, and follow-up 2: 50 ± 15 mL/min/1.73 m^2^).

## 4 Discussion

This study confirms that renal function decline in AF patients is approximately 1.3-fold greater than the physiological decline (van der Burgh A.C., et al., 2022). The main findings of this study are that the trajectories of renal function decline display a high variability in elderly AF patients and that the use of DOACs was significantly and independently associated with a smaller renal function decline compared to that observed in patients on WLDs.

As a matter of fact, the risk of WLDs-related nephropathy is particularly evident in patients with excessive anticoagulation (i.e., International Normalized Ratio (INR) > 3.0). These patients could experience glomerular and tubular hemorrhage, and approximately 30% of the patients manifest a rapid decline in renal function (eGFR loss >5 mL/min/1.73 m^2^) (Brodsky S.V., et al., 2011; Roldan V., et al., 2013; Wheeler D.S., et al., 2016; Violi et al., 2020). These results are in keeping with the subanalysis of the RELY trial for Dabigatran and the ANTENNA study for Rivaroxaban, as well as with the results that emerged in our observational study in which a better safety profile for renal function decline was found for all DOACs without Edoxaban compared to WLDs.

In the RELY sub-analysis (Bohm M., et al., 2015), the mean eGFR decline was significantly greater with warfarin than with Dabigatran (110 mg or 150 mg) after a mean follow-up of 30 months. Furthermore, the decline of renal function was more rapid in the group with poor INR control (i.e., time in the therapeutic range <65%).

The ANTENNA study compared the effect of rivaroxaban *versus* warfarin on renal outcomes over a 2.5-year follow-up period (Pérez A.G., et al., 2022). The mean eGFR decline was greater in patients of the warfarin group than in those of the rivaroxaban group (*p* = 0.03). Indeed, patients receiving DOACs compared to those on warfarin had a 37% reduced risk of the doubling of serum creatinine (HR: 0.63, CI 0.49–0.81), a 24% reduced risk of eGFR decline (HR: 0.76, CI 0.67–0.86), and a 23% lower risk of progression to end-stage renal disease (HR 0.77, CI 0.29–2.04) independently of type 2 diabetes mellitus (T2DM) or heart failure (HF). Another multicenter prospective observational study demonstrated that the median annual eGFR decline in patients of the WLDs group was worse than that observed in those on dabigatran, rivaroxaban, or apixaban (*p* = 0.003 vs. WLDs). However, in this paper, the median follow-up was rather short (1 year), and there were no data regarding Edoxaban (Pastori D., et al., 2020).

In the SCREAM (Stockholm Creatinine Measurements) project, 32,699 patients with AF were enrolled: 18,323 on DOACs and 14,376 on WLDs, with a median follow-up of 3.8 years (IQR 2.1–5.8) (Trevisan M., et al., 2022). This study shows that patients on DOACs had a 13% reduced risk of manifesting progression of CKD (HR 0.87, 95% CI 0.78–0.98) and a 12% reduced risk of manifesting acute kidney injury compared to those on WLDs (HR 0.88, 95% CI 0.80–0.97). However, patients enrolled in large randomized controlled trials were younger, with a lower burden of comorbidities, and were followed up for a shorter period compared to those enrolled in our study. In a large cohort of 21,170 patients, with a mean age of 66 years and followed up for 3.5 years, patients on DOACs had a 50% reduced risk of progression to stage 4 of CKD (HR 0.5, CI 95% 0.28–0.89; *p* = 0.02) compared to those on WLDs, and an 85% reduced risk of progression to end-stage renal disease (HR 0.15, CI 95% 0.08–0.32; *p* < 0.001) (Choi S.H., et al., 2022).

In contrast, a sub-analysis of the ARISTOTLE study revealed that treatment with apixaban compared with warfarin resulted in a worsening of renal function, but this had no effect on efficacy and safety, which were maintained across the spectrum of renal function. At 12 months, the median eGFR decline in the entire study population was −1.02 (IQR -6.72/4.52) mL/min/1.73 m^2^, and in 13.6% of patients, there was a decline in eGFR >20% from the baseline. The median decline in the apixaban group was −1.37 (IQR -1.59/-1.15) and in the warfarin group −0.96 (IQR -1.18/-0.74), *p* = 0.01 (Hijazi Z., et al., 2016). Similar results were observed in the sub-analysis of the ROCKET-AF study in which, after a mean follow-up of 2.3 years, a decline of Creatinine Clearance (CrCl) of −4 (IQR -12/-3) mL/min was observed in the rivaroxaban group and −3 (IQR -11/-4) mL/min in the warfarin group, *p* < 0.001 (Fordyce C.B., et al., 2016). The worsening of CrCl ≥20% was reported in 27% of patients of the rivaroxaban group and in 26% of those on warfarin, *p* = 0.09 (Fordyce C.B., et al., 2016). One observational study compared the safety of DOACs vs. WLDs on renal function decline over a mean follow-up of 3.3 ± 0.9 years and showed no statistically significant differences in renal function decline between the warfarin and DOACs-treated patients (Lee W.C., et al., 2021).

In our study, we analyzed changes in renal function during a long-term period and analyzed the difference between WLDs and DOACs according to the length of follow-up. We found a lower decline of renal function in patients of the DOACs group compared to those of the WLDs group and this was true during the medium and particularly the long-term period, suggesting that the benefit of using DOACs regarding the renal function increases over time. A novel analysis of our work consists of the application of trajectories analysis by which we demonstrated that patients of the DOACs group *versus* WLDs group were 3.03 to 4.24-fold more likely to belong to the trajectory with the smallest eGFR decline and this was also true after data adjustment for potential confounders.

These findings are biologically plausible and are related to the WLDs’ mechanism of action, pharmacokinetic and pharmacodynamic properties, and their relative lack of specificity. In fact, WLDs’ action is affected by numerous dietary and drug-drug interactions, vitamin K deficiency, and especially treatment compliance; these characteristics often result in reduced time in therapeutic range (TTR) with an increased risk of hemorrhagic or thromboembolic events. In contrast, DOACs, with equal efficacy of WLDs in the prevention of stroke and systemic thromboembolism, manifest a better safety profile especially in the elderly population (Roberti R., et al., 2021). In addition, WLDs decrease the carboxylation of matrix protein G1a, which is a major vitamin K-dependent inhibitor of medial and intimal vascular calcification and calciphylaxis, thus resulting in the progression of renal vascular calcifications that are associated with a decline in renal function and increased hemorrhagic and thromboembolic risks (Chatrou M.L., et al., 2012) (Aursulesei, 2019). Finally, patients with reduced TTR who manifest excess anticoagulation (i.e., INR >3.0) manifest anticoagulant-related nephropathy with further worsening of renal function due to thrombin depletion with glomerular hemorrhage and subsequent tubular obstruction with blood cylinders (Wheeler D.S., et al., 2016).

### 4.1 Limitations and strengths

Our study has limitations. The observational study design does not allow for definitively establishing a cause-effect relationship between the use of DOACs and renal function deterioration over time. Thus, our study is purely hypothesis-generating. Despite multiple measurements of eGFR during the time of the study, the overall sample size remains limited, with an imbalance in the number of patients between DOACs and WLDs. Another limitation of the study is represented by the absence of time in the therapeutic range of the patients treated with WLDs. Finally, the relatively low sample size precludes the possibility to investigate the effect of different DOACs categories (i.e., a drug-specific analysis) on the study outcome, an issue which should be formally tested in a future, adequately prepared study.

However, the study also has strengths including the enrolment of a population usually underrepresented in clinical trials such as the elderly with several comorbidities. Furthermore, patients enrolled were incident-users (or new users), thus eliminating the possibility of prevalent user bias related to previous WLDs exposition (Bohm M., et al., 2015). Finally, another strength was the long median follow-up period (4.9 years).

## 5 Conclusion

In elderly patients with AF, the use of DOACs was associated with a smaller decline of renal function over time compared to the treatment with WLDs, notwithstanding that patients in the DOACs group were older and with a higher burden of comorbidities. This is consistent with what was partly reported in the literature. Considering that CKD increases the risk of thrombotic and hemorrhagic events in patients with AF and that a physiological decline in eGFR occurs with aging, strategies aimed at preserving renal function are warranted. Therefore, in elderly patients with AF and several comorbidities, DOACs are preferred over WLDs to preserve renal function.

## Data Availability

The raw data supporting the conclusion of this article will be made available by the authors, without undue reservation.
