# Supplementary figures and images for: Medium-term and long-term renal function changes with direct oral anticoagulants in elderly patients with atrial fibrillation
Source: Front Pharmacol. 2023 Jul 4;14:1210560. doi: 10.3389/fphar.2023.1210560 (PMC10352777; doi:10.3389/fphar.2023.1210560)

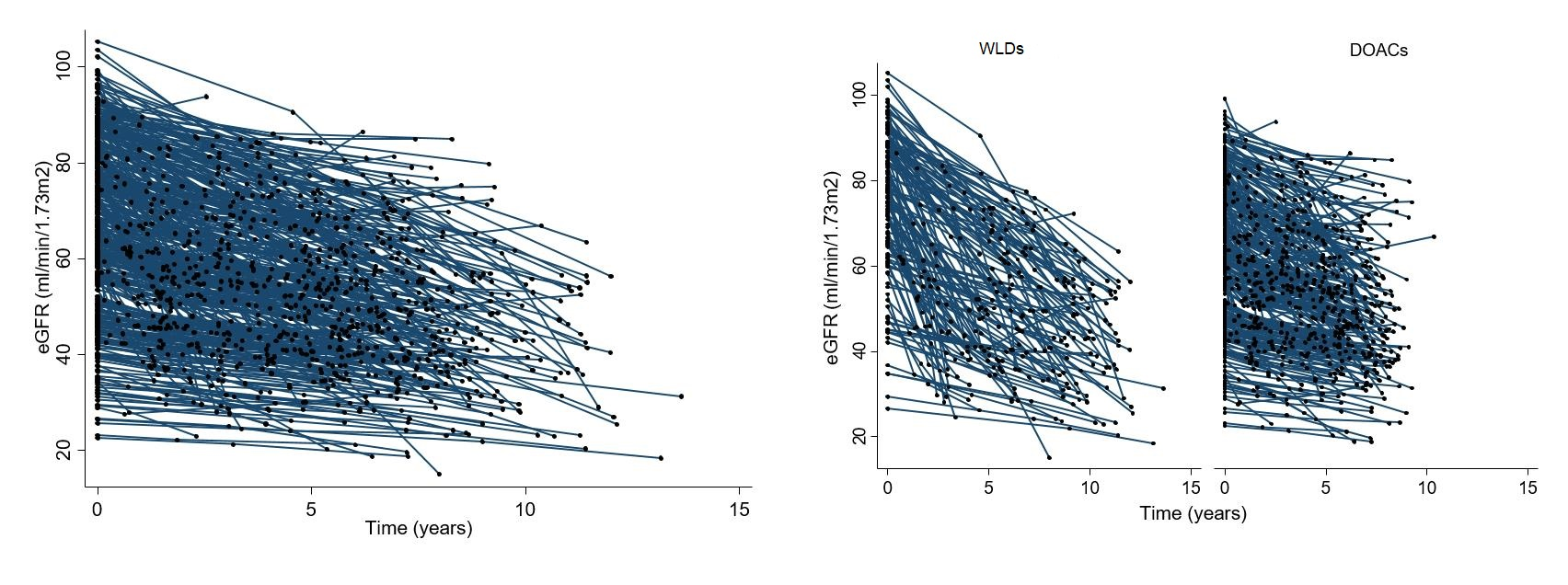

Supplement: Supplementary file 1 [file Image1.TIFF]
