# Supplementary material for: Medium-term and long-term renal function changes with direct oral anticoagulants in elderly patients with atrial fibrillation
Source: Front Pharmacol. 2023 Jul 4;14:1210560. doi: 10.3389/fphar.2023.1210560 (PMC10352777; doi:10.3389/fphar.2023.1210560)
Supplement: Supplementary file 2 [file DataSheet1.docx]

**APPENDIX**

*Trajectories analysis*

By GBTM analysis (Nagin D.S., 1999), patients were grouped according to the trajectories of their eGFR values over time. The eGFR measurements were modelled using the censored normal distribution for continuous data approximately normally distributed, with or without censoring (Jones B.L., et al. 2001). The trajectory analysis identified groups with similar eGFR trajectories based on maximum likelihood estimates. Each patient was assigned to the trajectory on the basis of the higher posterior probability. For the model selection, we considered parsimony, distinctive features of data, clinical judgment, and observed latent classes in practice. To establish the number of groups, we applied criteria based on the higher Bayesian information criterion (BIC) (Roeder K., et al. 1999). We considered the significance of polynomial terms starting with a quadratic specification for the trajectory shape and dropping non-significant polynomial terms (Andruff H., et al. 2009). Furthermore, to assess the adequacy of the final model, we investigated the group specific average posterior probability of group membership (>0.70 indicated a satisfactory model fit). Other criteria were as follows: the size of each group, the odds of correct classification higher than 5 based on the posterior probabilities of group membership for each trajectory group, and the strong correspondence between each group’s estimated probability and the proportion of study members assigned to that group basing on the maximum posterior probability (Nagin D.S., Odgers C.L. 2010). Entropy, ranging from 0.00 to 1.00, was considered a summary indicator of the conditional probabilities of individuals’ group membership: high values (>0.80) indicate that individuals are well classified and there is adequate separation between the latent classes (van der Burgh A.C. et al. 2022). The results of the trajectory were represented using the trajectory plot (each trajectory also includes 95% confidence intervals), and the percentage of patients in each group was given. Trajectory groups were ranked from the first to the fifth according to the eGFR loss (from the lowest to the highest) over time. For each trajectory, we reported the beta (i.e. the slope) and the standard error. The linear slope parameter estimate represents the amount of decrease of the eGFR over approximately 3.3 years. To investigate the associations between trajectory groups and baseline covariates we used χ^2^ for proportions, ANOVA and Kruskal-Wallis for normally and non-normally distributed variables. The level of statistical significance was p<0.05. Once trajectories had been made, we proceeded with a multinomial logistic regression to evaluate the relationship between trajectories and treatment, making crude and adjusted models. The reference group was the one with the greatest loss of glomerular filtrate over follow-up period. In multiple models we adjusted for all variables that significantly differed between the two treatment groups with the exception of age, hypertension and previous ACS (because these variables were already included into the CHA2DS2VASc score, i.e. a covariate into the same models). In these models, data were expressed as Probability ratio, 95% CI and p-value. Analyses were performed in STATA package (16.1 for Windows, TX United States).

**Supplementary Table 1 –Analytical details about the adequacy of GBTM analysis.**

| Trajectories  Groups | N | Posterior average  mean probability | OCC | Groups  percentage |
| --- | --- | --- | --- | --- |
| 1 | 25 | 0.90 | 126.76 | 0.06 |
| 2 | 96 | 0.89 | 26.45 | 0.23 |
| 3 | 135 | 0.88 | 15.74 | 0.32 |
| 4 | 96 | 0.92 | 38.80 | 0.23 |
| 5 | 68 | 0.79 | 19.39 | 0.16 |

**Supplementary Table 2 Estimated group probability and proportion assigned to group according to the maximum posterior probability assignment rule.**

| Trajectories | Estimated  group probability | Proportion assigned to group according  to the maximum posterior probability assignment rule |
| --- | --- | --- |
| 1 Group | 6.38 | 5.95 |
| 2 Group | 22.87 | 22.86 |
| 3 Group | 31.81 | 32.14 |
| 4 Group | 22.86 | 22.86 |
| 5 Group | 16.08 | 16.19 |

**Supplementary table 3 - Baseline characteristics of patients according to trajectories groups.**

|  | **Whole population**  **(N=420)** | **Trajectory 1**  **(N=25)** | **Trajectory 2**  **(N=96)** | **Trajectory 3**  **(N=135)** | **Trajectory 4**  **(N=96)** | **Trajectory 5**  **(N=68)** | **p** |
| --- | --- | --- | --- | --- | --- | --- | --- |
| **Demographic and clinical parameters** |  |  |  |  |  |  |  |
| Age, years | **77±6** | **79±4** | **79±5** | **77±5** | **74±6** | **75±6** | <0.001 |
| Gender (males), % | **55%** | **16%** | **44%** | **53%** | **77%** | **59%** | <0.001 |
| BMI, Kg/m2 | 29±4 | 29±4 | 29±4 | 29±4 | 29±4 | 30±3 | 0.55 |
| Waist, cm | 109±10 | 108±10 | 108±11 | 109±11 | 109±11 | 110±8 | 0.76 |
| Smokers, % | 9% | 12 | 2 | 13 | 10 | 9 | 0.08 |
| Systolic BP, mmHg | 133±12 | 130±17 | 133±10 | 132±14 | 133±10 | 133±9 | 0.86 |
| Diastolic BP, mmHg | 77±10 | 77±10 | 77±9 | 77±9 | 76±11 | 75±10 | 0.75 |
| Pulse Pressure, mmHg | 56±12 | 53±14 | 56±11 | 55±13 | 56±11 | 57±11 | 0.51 |
| Atrial Fibrillation, type % |  |  |  |  |  |  | 0.46 |
| Paroxysmal, *%* | 17% | 20% | 17% | 17% | 16% | 21% |  |
| Persistent, *%* | 17% | 8% | 13% | 15% | 21% | 24% |  |
| Permanent, *%* | 66% | 72% | 71% | 68% | 64% | 56% |  |
| PM or ICD, *%* | 9% | 16% | 6% | 9% | 6% | 15% | 0.20 |
| CHA_2_DS_2_VASc, *pt* | **4 (3-5)** | **4 (4-5)** | **4.5 (4-6)** | **4 (4-6)** | **4 (3-5)** | **4 (3-5)** | 0.02 |
| **Comorbidities** |  |  |  |  |  |  |  |
| Hypertension, % | 90% | 84% | 95% | 89% | 92% | 85% | 0.23 |
| Diabetes, % | 40% | 32% | 46% | 39% | 43% | 37% | 0.61 |
| Dyslipidaemia, % | 44% | 48% | 42% | 44% | 48% | 40% | 0.83 |
| Respiratory insufficiency/COPD, *%* | 40% | 36% | 43% | 41% | 35% | 38% | 0.83 |
| Heart failure, % | 34% | 24% | 35% | 33% | 35% | 34% | 0.86 |
| SAS, % | 28% | 32% | 34% | 24% | 27% | 28% | 0.49 |
| Type of SAS, % (N=118) |  |  |  |  |  |  | 0.98 |
| OSA, *%* | 40% | 25% | 36% | 41% | 42% | 47% |  |
| CSA, *%* | 52% | 63% | 58% | 50% | 50% | 42% |  |
| MSA, *%* | 8% | 13% | 6% | 9% | 8% | 11% |  |
| Severity of SAS, % (N=118) |  |  |  |  |  |  | 0.82 |
| Mild, *%* | 97% | 100% | 97% | 97% | 100% | 95% |  |
| Moderate, *%* | 3% | 0% | 3% | 3% | 0% | 5% |  |
| Severe, *%* | 0% | 0% | 0% | 0% | 0% | 0% |  |
| Liver disease, *%* | 21% | 20 | 17 | 23 | 22 | 22 | 0.82 |
| Cardiovascular background comorbidities |  |  |  |  |  |  |  |
| Previous STROKE/TIA, *%* | 11% | 0% | 11% | 11% | 9% | 85% | 0.35 |
| Previous ASC, *%* | 24% | 12% | 26% | 26% | 26% | 37% | 0.54 |
| Vasculopaties, *%* | 61% | 60% | 54% | 64% | 64% | 40% | 0.57 |
| **Drugs** |  |  |  |  |  |  |  |
| PPI, *%* | 91% | 92% | 91% | 93% | 90% | 88% | 0.77 |
| Nitrates, *%* | **7%** | **24%** | **6%** | **8%** | **5%** | **4%** | 0.018 |
| ACEi/ARBs, *%* | 81% | 76% | 86% | 79% | 80% | 81% | 0.63 |
| β-Blockers, *%* | 71% | 80% | 77% | 72% | 66% | 66% | 0.30 |
| Digoxin, *%* | 21% | 24% | 25% | 20% | 20% | 21% | 0.88 |
| Calcium-blockers, *%* | 15% | 24% | 18% | 11% | 16% | 16% | 0.44 |
| MRAs, *%* | 25% | 12% | 21% | 29% | 25% | 28% | 0.34 |
| ARNI, *%* | 33% | 24% | 34% | 33% | 35% | 32% | 0.87 |
| Anti-Arrhythmics Drugs, *%* | 17% | 8% | 16% | 15% | 17% | 25% | 0.27 |
| Metformina/OADs, *%* | 23% | 24% | 26% | 23% | 22% | 21% | 0.94 |
| Insulina, *%* | 12% | 24% | 17% | 13% | 9% | 6% | 0.08 |
| SGLT2i, *%* | 31% | 12% | 29% | 33% | 33% | 32% | 0.30 |
| Incretins, *%* | 40% | 32% | 45% | 38% | 44% | 37% | 0.61 |
| Lipid lowering drugs, *%* | 49% | 52% | 47% | 51% | 52% | 44% | 0.83 |
| VKAs, *%* | **32.4%** | **20%** | **23%** | **26%** | **41%** | **51%** | <0.001 |
| DOACs, *%* | **67.6%** | **80%** | **77%** | **74%** | **59%** | **49%** |  |
| VKAs, *%* | **32.4%** | **20%** | **23%** | **26%** | **41%** | **51%** | 0.005 |
| Dabigatran, *%* | **13.1%** | **24%** | **13%** | **13%** | **14%** | **9%** |  |
| Rivaroxaban, *%* | **34.3%** | **36%** | **39%** | **41%** | **30%** | **21%** |  |
| Apixaban, *%* | **11.9%** | **16%** | **11%** | **10%** | **13%** | **13%** |  |
| Edoxaban, *%* | **8.3%** | **4%** | **15%** | **10%** | **3%** | **6%** |  |
| **Biochemical parameters** |  |  |  |  |  |  |  |
| Albumin, *g/dl* | 3.90±0.35 | 3,82±0,35 | 3,92±0,36 | 3,91±0,36 | 3,93±0,32 | 3,85±0,38 | 0.423 |
| Total cholesterol, *mg/dl* | 168±44 | 164±55 | 164±44 | 172±45 | 165±39 | 172±43 | 0.57 |
| LDL cholesterol, *mg/dl* | 101±35 | 103±36 | 98±35 | 102±37 | 100±31 | 102±36 | 0.93 |
| HDL cholesterol, *mg/dl* | 52±18 | 53±25 | 53±17 | 52±17 | 52±20 | 52±16 | 0.99 |
| Triglycerides, *mg/dl* | 98 (72-137) | 106(79-138) | 94.5 (70-132) | 102 (72-149) | 88 (70.5-133) | 100 (76-128) | 0.51 |
| Na, *mmol/l* | 140.64±2.31 | 141,08±1,68 | 140,6±2,33 | 140,59±2,38 | 141,06±2,41 | 140,07±2,12 | 0.08 |
| Haemoglobin, *g/dl* | 13.31±1.53 | 13,01±1,38 | 13,26±1,5 | 13,24±1,58 | 13,51±1,49 | 13,32±1,61 | 0.56 |
| K, *mmol/l* | 4.41±0.37 | 4,38±0,39 | 4,25±0,41 | 4,46±0,38 | 4,48±0,32 | 4,42±0,31 | <0.001 |
| Creatinine, *mg/dl* | **1.00±0.28** | 1,64±0,27 | 1,23±0,22 | 0,98±0,13 | 0,79±0,13 | 0,78±0,11 | <0.001 |
| e-GFR, *ml/min/1.73m2* | **67.36±18.16** | 31,89±4,51 | 48,61±7,22 | 65,67±7,22 | 85,89±7,53 | 84,11±6,33 | <0.001 |
| NT- proBNP, *pg/ml* | 562 (512-1460) | 550 (478-632) | 598 (517.5-1361.5) | 598 (523-1656) | 632 (517-2139) | 548 (487-960) | 0.0168 |
| Fasting glucose, *mg/dl* | 103 (96-116) | 113 (100-126) | 105 (98-125) | 100 (93-119) | 102 (95-114) | 104 (97-114) | 0.131 |
| Fasting Insulin, *µU/ml* | 16.36±6.53 | 17,44±8,5 | 17,54±7,00 | 15,36±6,02 | 16,15±5,8 | 16,62±6,86 | 0.13 |
| HOMA, | 4.25 (2.96-5.72) | 4.97 (3.15-6.84) | 4.71 (3.22- 6.32) | 3.95 (2.69-5.34) | 4.30 (3.02-5.44) | 4.15 (3.04-5.70) | 0.10 |
| Uric acid, *mg/dl* | 5.61±0.96 | 5,5±0,94 | 5,64±0,94 | 5,68±1,01 | 5,62±0,92 | 5,45±0,95 | 0.56 |
| AST, *IU/L* | 21.17±7.72 | 19,08±5,97 | 20,95±7,59 | 21,65±9,1 | 21,16±6,19 | 21,29±7,48 | 0.65 |
| ALT, *IU/L* | 20.65±10.69 | 18,16±11,16 | 19,85±11,04 | 21,93±12,3 | 20,44±8,71 | 20,49±8,95 | 0.42 |
| Alkaline phosphatase, *IU/L* | 78 (66-110) | 86 (68-134) | 76 (66-105) | 77(64-107) | 76(66-111) | 75(66-112) | 0.65 |
| GGT, *IU/L* | 33 (21-44) | 30(16-38) | 34(21-43) | 33 (23-51) | 30(19-39) | 33 (17-44) | 0.39 |

**Abbreviations:** **VKAs,** vitamin K antagonists ; **DOACs,** direct oral anticoagulants; **BMI,** body mass index; **BP,** blood pressure; **PM,** pacemaker; **ICD,** implantable cardioverter defibrillator; **COPD,** Chronic Obstructive Pulmonary Disease; **SAS,** Sleep apnea syndrome; **OSA,** obstructive sleep apnea, **CSA,** central sleep apnea; **MSA,** mixed sleep apnea; **TIA,** transient ischemic attack; **ACS,** acute coronary syndrome; **PPIs,** Proton pump inhibitors; **ACEi**, angiotensin-converting enzyme inhibitor; **ARBs,** angiotensin receptor blockers; **MRAs,** mineralocorticoid receptor antagonists; **ARNI,** angiotensin receptor neprilysin inhibitor; **OADs,** oral antidiabetic drugs; **SGLT2i,** sodium-glucose cotransporter 2 inhibitor; **LDL,** low density lipoprotein; **HDL,** high density lipoprotein; **Na,** Sodium; **K,** Potassium; **e-GFR,** estimated glomerular filtration rate; **NT-proBNP,** N-terminal pro-brain natriuretic peptide; **HOMA,** homeostatic model assessment; **AST,** Aspartate Transaminase; **ALT,** Alanine Aminotransferase; **GGT,** gamma glutamyltranspeptidase.
